# Towards physiologically relevant human pluripotent stem cell (hPSC) models of Parkinson’s disease

**DOI:** 10.1186/s13287-021-02326-5

**Published:** 2021-04-29

**Authors:** Elena Coccia, Tim Ahfeldt

**Affiliations:** 1 Nash Family Department of Neuroscience, Icahn School of Medicine at Mount Sinai, New York, 10029 NY US; 2 Department of Neurology, Icahn School of Medicine at Mount Sinai, New York, 10029 NY US; 3 Friedman Brain Institute, Icahn School of Medicine at Mount Sinai, New York, 10029 NY US; 4 Ronald M. Loeb Center for Alzheimer’s Disease, Icahn School of Medicine at Mount Sinai, New York, 10029 NY US; 5 Black Family Stem Cell Institute, Icahn School of Medicine at Mount Sinai, New York, 10029 NY US

**Keywords:** Neurodegenerative disease modeling, Human pluripotent stem cells (hPSCs), CRISPR, Parkinson’s disease, *GBA*

## Abstract

The derivation of human embryonic stem cells followed by the discovery of induced pluripotent stem cells and leaps in genome editing approaches have continuously fueled enthusiasm for the development of new models of neurodegenerative diseases such as Parkinson’s disease (PD). PD is characterized by the relative selective loss of dopaminergic neurons (DNs) in specific areas of substantia nigra pars compacta (SNpc). While degeneration in late stages can be widespread, there is stereotypic early degeneration of these uniquely vulnerable neurons. Various causes of selective vulnerability have been investigated but much remains unclear. Most studies have sought to identify cell autonomous properties of the most vulnerable neurons. However, recent findings from genetic studies and model systems have added to our understanding of non-cell autonomous contributions including regional-specific neuro-immune interactions with astrocytes, resident or damage-activated microglia, neuro-glia cell metabolic interactions, involvement of endothelial cells, and damage to the vascular system. All of these contribute to specific vulnerability and, along with aging and environmental factors, might be integrated in a complex stressor-threshold model of neurodegeneration. In this forward-looking review, we synthesize recent advances in the field of PD modeling using human pluripotent stem cells, with an emphasis on organoid and complex co-culture models of the nigrostriatal niche, with emerging CRISPR applications to edit or perturb expression of causal PD genes and associated risk factors, such as *GBA*, to understand the impact of these genes on relevant phenotypes.

## Limitations of PD models

Parkinson’s disease (PD) is a neurodegenerative disorder (Table 1) for which the exact pathogenesis is still unknown, and no disease-modifying treatment exists to date. It is therefore essential to refine research models to better understand the pathophysiology underlying the neuronal loss and to develop therapeutic strategies. Postmortem studies of PD patients have laid the foundation of our understanding of pathological aspects of the disorder. These include the presence of Lewy bodies, inclusions primarily composed of aggregated α-synuclein (α-syn), regional vulnerability of dopaminergic neurons, and the impact of oxidative stress, inflammation, infections, and environmental toxins [10]. However, these studies are restricted by the rarity of brain samples, ethical limitations, the loss of the most vulnerable neurons, and the restricted temporal window in which to examine samples, which has hindered genetic and mechanistic studies.

**Table 1:** Parkinson’s disease

﻿Parkinson’s disease (PD) is a complex, progressive neurodegenerative condition affecting more than 1% of the population over 65 years of age. By the time clinical symptoms appear, around ~ 50% dopaminergic neurons in the substantia nigra pars compacta (SNpc), are lost. Most PD cases exhibit abnormal intracellular protein aggregates called Lewy bodies. These are composed largely of aggregated α-synuclein (α-syn). With the progression of the disease, the neurodegeneration in this region reaches 90%, while dopaminergic neuron loss in the dorsal tier may be as low as 25%, and many other brain regions are relatively unaffected [1]. The striatal selective neurodegeneration is clinically characterized by debilitating motor and non-motor features which cause severe disability [2]. About 10% of PD cases are monogenic forms, associated with highly penetrant gene mutations [3, 4]. However, the majority of PD cases are sporadic, with a complex etiology caused by an interplay of environmental and associated genetic risk factors. Over the last few years, large scale genome-wide association studies (GWAS) and meta-analyses have identified more than 90 loci of different frequency and penetrance associated with sporadic PD risk. Most of the loci confer a small PD risk individually, but have shown to possess a substantial cumulative risk [5–9].	

Animal models have been extensively used for PD research. While parkinsonism is frequently induced by acute toxins, more recently transgenic animal models of familial PD genes have been developed. Although these models have provided a better understanding of in vivo mechanisms of PD pathogenesis, they are often limited by a lack of specific neuronal degeneration and fail to recapitulate the progression of the disease and features of movement disorders [11–13]. Researchers have experienced difficulties in translating promising findings from animal models into successful trials in patients, mainly due to species differences in metabolism and in the sequence, pathogenicity, or number of isoforms expressed of key molecules [14–16]. As an appropriate model is fundamental for research and therapeutic development, the emergence of human pluripotent stem cell (hPSC) technology holds great promise to overcome these limitations and recapitulate essential aspects of the homeostatic and pathological alterations of disease-relevant cells in a human genetic background.

## Overview of the current PD models using hPSCs

hPSCs include blastocyst-derived human embryonic stem cells (ESC) [17] and induced-pluripotent stem cells (iPSC), which are reprogrammed from human somatic cells [18]. hPSCs are an innovative and unique alternative mean to model neuronal disease in vitro, as they have the potential to differentiate into multiple neuronal subtypes, as well as glial cells.

Various informative reviews have summarized PD models generated using patient-derived iPSCs- or, more recently, models created by gene-editing technology [19–24], although most are limited to DN modeling and the cell autonomous mechanism of degeneration. In this review, we will introduce new approaches that have greatly improved hPSC disease modeling, thanks to the development of specific protocols of differentiation, recreation of cellular interactions with co-culture experimental paradigms, and 3D cellular systems.

The self-organizing capability of hPSCs permits the creation of three-dimensional aggregates, known as organoids because of their ability to recapitulate cell-cell interactions, cellular diversity, and structures found during organ development [25, 26]. Brain organoids are especially advantageous for modeling adult-onset diseases, as they allow for the differentiation and maturation of neurons and for long-term cultures, in which disease-associated phenotypes can be promoted through multiple or chronic treatments. Organoids enable the inclusion and complex interaction of different cell types in a spatially organized environment, and the possibility to elucidate the contribution of each cell type to a phenotype [27].

Midbrain organoids have been used to study the pathological mechanisms of PD-related gene mutations and have shown the ability to recapitulate hallmarks of the disease [28, 29]. These studies focused on relatively early time-points and on DNs, not considering cell populations that emerge at later time-points in organoid differentiation, such as astrocytes or oligodendrocytes. The integration of glia to the midbrain organoids would expand the potential of the model, enabling investigation of PD molecular dysregulation, neuro-glia interactions, and neuroinflammation, overall covering each of the main pathways currently implicated in PD pathology.

Among the cell types interacting with DNs, glia have been shown to be critical to faithfully model the in vitro key features of neurodegeneration pathology [30, 31]. Astrocytes and microglia are the two main types of glia. Their specific functions and cross-talk are actively involved in maintaining brain microenvironments [32, 33]. They strictly control neuronal homeostasis through metabolic support, phagocytic function, removal of apoptotic neuronal cells, and inflammatory response with cytokine production [34, 35]. In an environment of protracted stress, such as in neurodegenerative disease, glia divert from their beneficial function to take an active role in disease progression. Proinflammatory cytokines released from activated glia can become neurotoxic, enhance protein aggregation, and facilitate disease spread through transmission of protein aggregates to neurons via exosomes [36–39].

While initial PD research focus concentrated on DN intrinsic neuronal dysfunction, the contribution of glia in the pathology progression and development is now being investigated and recognized as decisive. Recently, astrocyte dysfunction has been directly involved in pathological mechanisms contributing to the degeneration of DNs [40, 41], and impacting neuronal levels of α-syn [42]. Co-culture systems of hPSC-derived cells have shown that familial PD mutations in *LRRK2* and ATP13A2 (*PARK9*) induce astrocytic functional alterations, which results in a pathogenic crosstalk between astrocytes and neurons. Astrocytes harboring these PD mutations exhibit dysfunctional autophagy-lysosomal pathways, which induced toxic propagations of α-syn in DNs and neurodegeneration [42, 43]. Microglia are the main cell type responsible for the inflammatory response in the brain. Increasing evidence associates microglia-mediated responses with neurodegeneration in PD. Several loci found to be involved in microglia and innate immunity functions have been correlated to PD heritability [44–47], and elevated levels of pro-inflammatory cytokines and reactive microglia in the vicinity of dopaminergic neurons in the SN have been found in postmortem brain samples [48].

## Modeling PD in the engineered nigrostriatal niche

An increasing number of findings implicate numerous processes in the mechanism leading to neurodegeneration in PD, both in degenerating DNs (cell autonomous) and in other cell types (non-cell autonomous). A multi-cellular co-culture model appears to be essential to better understand the role of PD genes in the pathophysiology of the disease.

Various region-specific human brain organoids have been developed through distinct protocols of differentiation based on knowledge of brain development during embryology [49]. A recent step forward in disease modeling has been the ability to combine different region-specific pattered organoids into “assembloids” [50–53]. Assembloids bring another level of complexity to modeling, as cells generate a complex microenvironment which mimics essential in vivo aspects, including inter-regional interactions of differentially patterned cells, formation of neuronal circuits among neuronal subtypes, long-range axonal connections and cell migration [53, 54]. They provide a system in which it is possible to compare the effects of perturbations on selective susceptibility among regions affected and not affected by disease [55]. Furthermore, non-neuronal cell types like microglia or pericytes can also be engineered into assembloids, enabling the integration of vascular and immune systems in the model [56, 57].

**Fig. 1:**
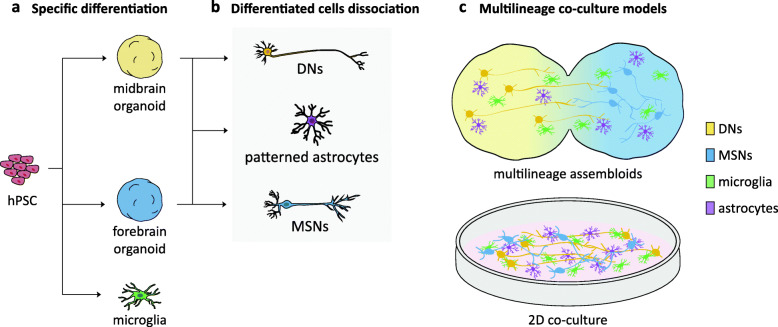
Modeling the nigrostriatal niche. Schematic representation of nigrostriatal multilineage co-culture models. **a** Human pluripotent stem cells (hPSC) undergo specific protocols of differentiation and generate region-patterned organoids (midbrain in yellow; forebrain in blue), as well as microglia (green). **b** Organoids dissociation allows for the isolation of neuronal populations and patterned astrocytes for further analysis. Dopaminergic neurons rise from the midbrain organoid (DNs, yellow), medium spiny neurons from the forebrain organoid (MSNs, blue), and region-patterned astrocytes from either one (purple). **c** hPSC-derived components are engineered into co-culture models. Midbrain and forebrain organoids are assembled and integrated with microglia to generate a multilineage assembloids model, which allows for neuronal circuit formation and neuro-immune communication in a 3D environment. Dissociated components are combined in 2D co-culture, a model in which each component can undergo differential genomic editing

We propose to combine existing differentiation technologies to engineer the nigrostriatal niche using two different multi-lineages culture models composed of defined neuronal and glial cell types relevant in PD (Fig. 1). Midbrain and forebrain organoid differentiation generates DNs and forebrain medium spiny neurons (MSNs) respectively. Region-patterned glial cells, such as astrocytes and oligodendrocytes arise after extended culture times [58]. Unlike cells derived from neuroectoderm, microglial cells are not present during brain organoid differentiation [59, 60], but can be generated separately and later integrated in the co-culture [30, 51, 61]. The fusion of midbrain and forebrain organoids seeded with microglia would create a complex nigrostriatal cellular environment and a physiologically relevant context to study PD. DNs would be able to establish long-range connections, complex interactions, and rely on glial support to form functional connections to forebrain synaptic targets [62]. Glia may establish neuro-immune communication and improve neuronal maturation [63–66].

Patterned brain organoids can be dissociated, and specific cell types can be combined in 2D co-culture. Even though the 2D system loses complexity compared to assembloids, it presents a stable population of cells over time and provides the opportunity to generate fully mixed-genetic models, as distinct cell types can be isolated and mixed from organoids with unique genetic perturbations. Neuro-immune interactions can be dissected among microglia, astrocytes, and neurons in long-term cultures. Both models offer a promising experimental paradigm in which to reproduce the complexity of the disease and explore the role of PD genes in the sequence of aberrant cellular functions at the basis of cell-type-specific neurodegeneration.

## Using advanced PD models to understand the role and function of risk genes

PD genetics are complex and despite recent advances, the majority of identified PD risk variants’ contribution to disease pathogenesis is poorly understood. More than 90 PD-related variants have been identified in the most recent GWAS. For most loci, it is still not known which genes underlie PD risk [8, 9, 67], as each locus identified encompasses hundreds of SNPs, and most lie within non-coding regions of the genome, potentially affecting the regulation of a network of nearby or distant coding genes [68, 69].

In order to understand how genetic variation leads to phenotypic differences, current genetic approaches are aimed at (i) identification of additional disease-associated SNPs/loci, (ii) fine mapping the causal SNPs and the genes impacted by variants, and (iii) understanding the specific effects of variants on cell types relevant to the pathology, or the combined effects in different cell types.

Several analytic strategies are being used to link specific GWAS loci to the causal genes that influence PD susceptibility [70, 71]. Quantitative trait loci (QTL) mapping correlates variants in genotype and changes in gene expression [72, 73]; the integration of previous genomic and functional annotation, single-cell RNA [74] or transcriptomic data [75] permits the determination of candidates’ expression in pathologically relevant cell populations or physiological contexts.

**Fig. 2:**
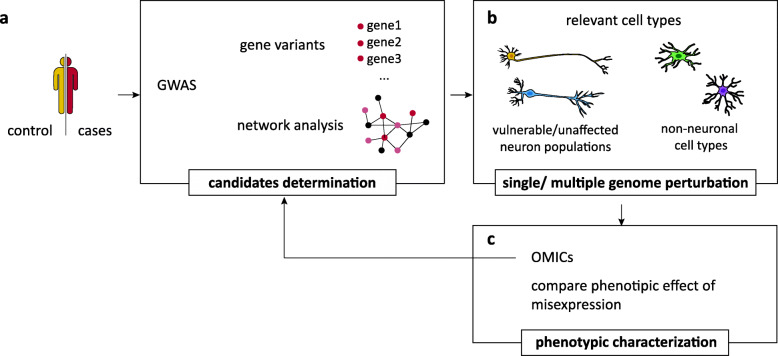
Pinpoint disease-relevant genes and cell types. **a** Samples collected from unaffected controls and patients are analyzed through genome-wide association studies (GWAS). Genetic variants determination and network analysis reveals genetic candidates. **b** To validate candidates’ effects, genome editing is carried out in disease-relevant cell types with isogenic background, as vulnerable and resilient population of neurons and glial cells. **c** Phenotypes are characterized and compared, in order to link functional consequences to specific genes and cell types. OMICs techniques allow to link candidate editing to a differential expression set of genes, overall generating additional candidates. Comparisons among different cell types elucidate genes responsible for cell-type-specific response and cell population vulnerability. Convergent phenotypes of differential genes pinpoint to pathology-relevant pathways. Multiple gene perturbations are useful to detect phenotype modifiers, that can either alleviate, therefore be considered potential therapeutic targets, as well as aggravate phenotypes

Once high-throughput studies prioritize candidate genetic variants related to the disease, the integration of gene editing tools such as CRISPR can be used to validate causal variants and genes and pinpoint the effect on disease-related phenotypes (Fig. 2). CRISPR genome editing allows for reengineering of the genome, epigenome, and transcriptome of cells [76]. The combination advances in both gene editing and hPSC technologies provide a powerful tool to test cell-type-specific effects of candidate genes and variants in isogenic models (reviewed in [20, 77, 78]). The strictly controlled and consistent genomic background enables the identification of even subtle in vitro phenotypes [79–81] and will be useful in efforts to stratify PD on a molecular level and identify a common therapeutic angle. In our lab, we used isogenic models to compare three distinct early-onset autosomal recessive forms of PD through CRISPR-mediated knockout (KO) of *PARKIN*, *ATP13A2*, and *DJ-1*. We observed a significant loss of DNs in *PARKIN*-KOs and showed dysregulation of the main pathways involved in PD [82] in all PD lines, including common mitochondrial dysfunction as well as lysosomal dysregulation and oxidative stress [29]. Taking these steps further, the recent iPSC Neurodegeneration Disease Initiative (iNDI) project is the largest genome editing project focused on Alzheimer’s Disease and related dementias, including a series of PD-causing mutations and as genetic risk factors such as *GBA*. These lines will be CRISPR edited into hPSC lines from unaffected individuals. iNDI will provide high-quality, deeply characterized isogenic iPSC lines that will be freely shared across the research community. Once available, this resource will greatly extend our ability to test common dysregulations in PD and our understanding of the related pathology [83].

In the past few years, CRISPR-mediated gene editing of PD genes has been successfully applied for many purposes in hPSC-derived models (reviewed in [84]). Phenotypes induced by autosomal dominant PD mutations have been explored by CRISPR-mediated correction and phenotype rescue. For example, the removal of two alleles in *SNCA* triplication iPSCs showed lower levels of α-syn aggregation and stress response [85]. Correction of A53T *SNCA* mutation has shown α-syn aggregation and lysosomal dysfunction associated with the pathogenic form [86]. LRRK2 G2019S point mutation, which is estimated to account for 1% of sporadic and 4% of familial PD patients [87], has been explored in several models. Mutation correction both in neurons [88] and astrocytes [43] as well as the introduction of the mutation in midbrain organoids [28], has implicated it in lysosomal dysfunction, α-syn accumulation, and neuronal cell death. In 2016, CRISPR was used to elucidate the effect of a common PD genetic risk variant at the *SNCA* locus on a molecular level. The risk-associated SNP, found in a non-coding distal enhancer element, was shown to significantly alter the expression of α-syn [89].

CRISPR-KO of several PD genes has pointed to the pathogenesis associated with their misexpression. A study using loss of DJ-1 models connected this mutation to commonly dysregulated PD pathways involving elevated α-syn, dysregulated *GBA*, as well as mitochondrial and lysosomal dysfunction and showed that oxidized dopamine may be part of the pathology. It was suggested that aspects of the observed pathology were unique in human models and not seen in mouse models which has been attributed to differences in dopamine metabolism [16].

Several technical challenges still remain. It is important to note that hPSCs are prone to genomic instability, abnormalities, or chromosome aberration, especially during long-term cultures or upon transgene transfection [90–92]. Moreover, genome editing may also cause unwanted edits, off-target effects, or transgene silencing upon hPSC differentiation [93]. Genome editing technology is in continuous advancement and the identification and application of new CRISPR effectors is constantly improving editing specificity, efficiency, genome stability, and target accuracy [94, 95].

Recent efforts in the scientific community have generated collaborative and international programs, such as the Global Parkinson’s Genetics Program (GP2) that will genotype more than 150,000 volunteers from a different ethnic background, or the Parkinson’s Progression Markers Initiative (PPMI) that will collect longitudinal clinical data and biosamples, including iPSCs generated from patients [96]. The availability of a robust and accurate PD model will be essential to maximize these resources to clarify PD genetic architecture and pathogenic pathways. The nigrostriatal niche, built on the conjunction of genome editing and multi-lineage modeling, offers a system in which to consider and compare gene perturbation among vulnerable and non-vulnerable populations of neurons. Each component of the model can undergo a different genetic edit, to recognize cell-specific effects of pathological gene mutations. Moreover, it holds promising advantages for future research to translate GWAS findings into cell-type-specific phenotypes as the identification of new CRISPR systems is increasing the ability to manipulate simultaneously several genes and multiple SNPs [97, 98].

## Modeling *GBA*, the major genetic risk factor in PD

A clear connection has been established between PD and the alteration of lysosomal pathways [99, 100]. Several familial PD genes as well as identified risk factors for sporadic PD converge in the function of this organelle. Among these, *GBA* is the most common genetic risk factor for PD and provides an ideal target for the nigrostriatal experimental model (Table 2). *GBA* is expressed in all cell types and encodes the lysosomal enzyme GCase. A decrease in GCase activity results in accumulation of its substrate, glucocerebroside, and compromised activity of the autophagy-lysosomal pathway [116]. The consequences of GBA mutations and GCase loss of function have been strongly linked to several pathological processes and hallmarks of PD in hPSC-derived midbrain DNs. GCase loss of function and lysosomal alteration compromise protein degradation, elevated α-syn levels and toxic buildup of aggregates in DNs [111, 117–120]. Glycosylceramide accumulation resulting from reduced GCase has been linked to several pathological pathways: deficit in autophagic flux, calcium imbalance [121], endoplasmic reticulum stress [122], and reduced dopamine storage and uptake [119, 123, 124]. iPSC-derived neurons from GD patients have shown several electrophysiological abnormalities, such as defects in action potential firing. Treatment of control iPSC neurons with a GCase inhibitor results in a similar phenotype, implicating GCase activity in the observed abnormal neuronal electrophysiological properties [125]. GCase activity has been suggested to play a role in PD pathogenesis even in the absence of *GBA* mutations. Other PD-related mutations, such as deficiency of the late endolysosomal transporter ATP13A2 (*PARK9*) have been recently shown to alter lysosomal function and decrease GCase activity in both DNs and astrocytes [42]. Additionally, accumulation of α-syn aggregates inhibits lysosomal maturation and activity of normal GCase, giving rise to a self-propagating bidirectional pathogenic loop that eventually leads to neurodegeneration [126, 127].

**Table 2:** The complex role of *GBA* in PD pathology

The *GBA* gene encodes the lysosomal enzyme glucocerebrosidase (GCase), responsible for the hydrolysis of glucocerebroside to glucose and ceramide [101]. Biallelic mutations in *GBA* result in GCase deficiency and cause Gaucher disease (GD), an autosomal recessive systemic lysosomal storage disorder with a complex pathogenesis [102, 103]. Heterozygous mutations in *GBA*, have been found to increase the risk of developing PD by 20–30-fold and are more than 5 times more likely to be found in PD patients compared to controls [104–106]. Clinical observations show that *GBA-*associated PD may have an earlier onset and higher risk of developing non-motor symptoms such as dementia and depression [107, 108]. Although the role of *GBA* mutations as risk factors for PD is well established, the mechanism underlying *GBA*-associated PD is still not clear. Glucocerebrosidase has a complex biology. The majority of *GBA* mutation carriers do not develop PD, and there is great variability in symptoms and clinical presentations in both GD and PD patients carrying the same mutations, suggesting the existence of modifying factors such as gene co-variants and environmental factors [109–111]. Decreased GCase activity is also found in patients with PD without *GBA* mutations, suggesting a central role of the enzyme in the pathogenesis of PD [112, 113]. Many different *GBA* mutations have been linked to PD disease including those resulting in null alleles and structural protein alteration that can affect stability or trafficking to the lysosome, as well as changes not related to enzymatic activity [114, 115].	

Studies of *GBA* have mainly focused on dysfunction in isolated cell types, which fails to model the complex interplay between other factors contributing to phenotype. *GBA* is expressed in all cell types, including glia [40, 128, 129], and is involved in essential processes for these cells, such as neuroimmune response and phagocytic activity [130, 131]. The first study considering the role of *GBA* mutation in glia with a human genetic background clearly suggests that astrocytes with mutant *GBA* may contribute to PD pathology. Residual levels of GCase activity in iPSC-derived astrocytes generated from GD patients appear to determine the degree of astrogliosis, inflammatory response, and ability to process α-syn [132].

**Fig. 3:**
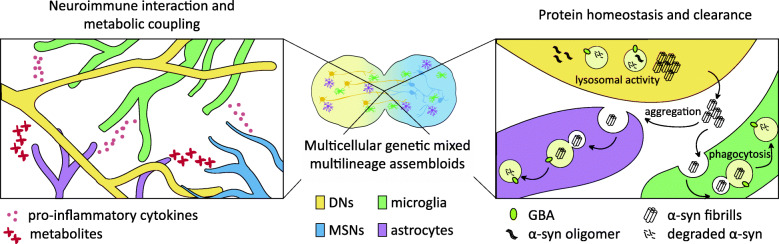
Modeling *GBA* biology in the nigrostriatal niche**.** Schematic representation of co-culture modeling for *GBA* study. The model presented can be used to determine cell-type-specific phenotypes associated with *GBA* misexpression. Astrocyte (purple) and microglia (green) strictly control neuronal homeostasis. Co-culture models allow for the assessment of neuroimmune interactions, metabolic support, and inflammatory response (left panel) as well as phagocytic and aggregates-clearance function (right panel). The model opens the possibility to perform the study in a physiologically relevant environment and evaluate how GCase dysfunction in a specific cell type impacts other components of the model

Although *GBA* has been linked to lysosomal functionality in isolated neuronal and glial cells, existing methods fail to resolve the relationship between *GBA* and PD pathogenesis, as they do not explain cell type vulnerability and incomplete penetrance of *GBA* variants. The nigrostriatal multilineage assembloid model offers an innovative complex co-culture paradigm that overcomes previous limitations to our understanding of the complexity of gene-variant induced phenotypes. Isogenic hPSC cell lines can be differentiated into glia, susceptible or unaffected neuronal populations, and combined in genetically mixed co-culture to dissect cell-type contributions to pathological phenotypes (Fig. 3). Using high-density SNP arrays different genes have been reported to influence *GBA*-associated risk of disease [133] and could partially explain the observed low penetrance. Emerging CRISPR system tools enable us to simultaneously target multiple genes and evaluate how different variants with individually small effect may synergistically affect *GBA* pathways and aggravate or ameliorate PD phenotypes [97, 134].

## Extending the complex hPSC models to study other neurodegenerative diseases

Neurological disorders are the leading source of disability globally [135]. A common clinical feature shared among different neurodegenerative diseases is the presence of protein-based deposits as intracellular inclusions and/or as extracellular aggregates, present in specific neuronal types in distinct regions of the brain [136–139] (Table 3). Genetic studies and experimental findings have shown that both genetic and environmental factors impact the onset and progression of the pathology [140, 141].

**Table 3:** Neurodegenerative disease unique protein aggregates and neuronal susceptibility

	Protein inclusion	Most vulnerable neuronal population
Alzheimer’s disease	Neurofibrillary tangles (phosphorylated tau); amyloid plaques (amyloid β peptide)	Pyramidal neurons (entorhinal cortex, hippocampus, locus coeruleus)
Parkinson’s disease	Lewy bodies (α-synuclein)	DA neurons (substantia nigra pars compacta)
Huntington’s disease	Aggregated huntingtin (mutated huntingtin)	MSN neurons (striatum)

In most cases, the proteins and genes implicated in the etiology of these diseases have a widespread, ubiquitous expression; nevertheless, the degeneration selectively targets specific neuronal types and regions. A major unresolved question in the field is the mechanism underlying the selective vulnerability of specific neuronal subpopulations.

The complexity of neurodegenerative diseases requires new methods to understand the specific dysfunction and multifactorial origins contributing to disease pathogenesis. With the recent development of specific differentiation protocols [142–144], multicellular assembloid models can be extended to study the mechanisms involved in other neurodegenerative diseases in which single gene differences can point to pathological mechanisms in a specific cell type, but mostly do not explain cell type vulnerability. For example, Huntington’s disease is characterized by widespread cell death in the striatum of carriers of the mutant huntingtin gene. The most vulnerable and first neurons to degenerate are MSNs of the striatum, while other regions are unaffected [145]. The molecular basis underlying this highly specific neurodegeneration is still not clear. The nigrostriatal model proposed could be used to model huntingtin gene mutation in MSNs and others cell types in order to clarify the possible involvement of non-autonomous cell functions.

Multi-lineage assembloids are a scalable system in which to integrate several variables and risk factors related to neurodegenerative diseases simultaneously. The model offers a novel experimental paradigm which takes complexity and phenotypic heterogeneity into consideration [146]. Understanding the basis for cell type vulnerability and the factors involved in disease pathogenesis will provide insights regarding how to specifically intervene to reverse cellular dysfunction, support the affected cell types, and enable the development and testing of future therapeutics.

## Data Availability

Not applicable, no data were generated in the study.

## Abbreviations

α-syn: α-Synuclein
DNs: Dopaminergic neurons
GCase: Glucocerebrosidase
GD: Gaucher disease
GWAS: Genome-wide association studies
hESCs: Embryonic stem cells
hPSCs: Human pluripotent stem cells
iPSCs: Induced PSCs
MSNs: Medium spiny neurons
PD: Parkinson’s disease
QTL: Quantitative trait loci
SNpc: Substantia nigra pars compacta
